# From Knowledge to Differentiation: Increasing Emotion Knowledge Through an Intervention Increases Negative Emotion Differentiation

**DOI:** 10.3389/fpsyg.2021.703757

**Published:** 2021-11-26

**Authors:** Evgeniya Vedernikova, Peter Kuppens, Yasemin Erbas

**Affiliations:** ^1^Faculty of Psychology and Educational Sciences, KU Leuven, Leuven, Belgium; ^2^Tilburg School of Social and Behavioral Sciences, Tilburg University, Tilburg, Netherlands

**Keywords:** emotion, emotion differentiation, emotional granularity, emotion knowledge, emotion components, emotion intervention

## Abstract

Labeling emotions with a high degree of granularity appears to be beneficial for well-being. However, there are individual differences in the level of emotion differentiation, and some individuals do not appear to differentiate much between different emotions. Low differentiation is associated with maladaptive outcomes, therefore such individuals might benefit from interventions that can increase their level of emotion differentiation. To this end, we tested the effects of an emotion knowledge intervention on the level of emotion differentiation. One hundred and twenty participants were assigned to either an experimental or a control condition. Emotion differentiation was assessed with a Scenario Rating Task before and after the intervention, and at follow-up. As predicted, negative emotion differentiation increased significantly after the emotion knowledge intervention, and this increase was not observed in the control group. Positive emotion differentiation also increased slightly; however, it did not reach significance level. This finding suggests that an emotion knowledge intervention might be beneficial for increasing negative emotion differentiation and may have implications for the clinical context.

## Introduction

Emotions are ubiquitous in our lives. Individuals experience emotions every day, in response to both minor events and to significant moments of their lives. Emotions can make one feel sky-high or, the opposite, extremely low. Emotions experienced in response to events can help to navigate these events by providing information about them, which can in turn help people to deal with the situation. Given this function, emotions are considered necessary for effective adaptation (Smith and Lazarus, 1990), which in turn is essential for mental health (Manwell et al., 2015).

An important process, through which emotions can be used adaptively, is emotion differentiation, also called as emotional granularity. Emotion differentiation is defined as the tendency to distinguish among one’s own emotions and to label one’s emotions in a discrete way, sensitive to context (Barrett et al., 2001; Quoidbach et al., 2014; Kashdan et al., 2015). For instance, when asked about their feelings in response to different events, a low differentiator might report feeling both sad and anxious across all situations, whereas a high differentiator would report different emotions in different situations, for example, sad and guilty in response to one event, and anxious, overwhelmed and disappointed in response to another one.

Emotion differentiation is found to be related to various indicators of well-being. For instance, negative emotion differentiation was related to lower levels of negative emotion intensity, depression, neuroticism, and to higher levels of self-esteem (Erbas et al., 2014; Willroth et al., 2019). It also weakened the relationship between rumination and depression (Liu et al., 2020; Seah et al., 2020) as well as the relationship between negative emotions and decreased intrinsic motivation (Vandercammen et al., 2014). In adolescence, negative emotion differentiation was related to lower negativity intensity and negativity propensity (Lennarz et al., 2018). Furthermore, emotion differentiation also appeared to facilitate more successful emotion regulation (Barrett et al., 2001; Kalokerinos et al., 2019). For instance, higher levels of emotion differentiation protected individuals from destructive behavior such as excessive alcohol consumption (Kashdan et al., 2010), aggression (Pond et al., 2012), and unhealthy eating behavior (Mikhail et al., 2019). Positive emotion differentiation in turn was associated with more effective coping styles, i.e., less mental self-distraction during stressful times, higher engagement in the coping process, less automatic responding, and greater thinking through behavioral options before acting (Tugade et al., 2004). Higher differentiation also appeared to be beneficial in relationships with others: it was related to more empathic accuracy (Erbas et al., 2016) and better recognition of others’ emotional expressions (Israelashvili et al., 2019). Together, these studies suggest that high levels of emotion differentiation have important implications for well-being.

One factor that may underlie between-person differences in the level of emotion differentiation is related to the amount of unique information an individual associates with each emotion construct. It appears that individuals differ in how different emotion labels are associated with different multimodal instances of affect (Gohm and Clore, 2000). High differentiators link very specific information about the situation (e.g., behavior and physiological response) to particular emotion labels, whereas low differentiators, in contrast, link different labels to more similar and overlapping patterns of such elements (e.g., Erbas et al., 2015).

Consequently, a possible reason why emotion differentiation is beneficial for well-being is because a more granular way of labeling emotions may indicate that individuals represent the unique aspects of emotional events in a highly specific way (Erbas et al., 2018). Thus, when individuals can differentiate their emotions (not disgust, not anger, but fear), they access the information those emotions entail regarding the environment and/or circumstances (e.g., the environment is dangerous; Kashdan et al., 2015; Kalokerinos et al., 2019), which is more specific for individuals with more granular emotions. When this information is perceived and processed, individuals can then use this information to regulate their emotions in order to facilitate strivings (e.g., there is no time for extensive fear and panic, an action is needed).

However, in order to be able to represent aspects of the emotional event with a level of specificity that can be used in a context-sensitive way, different characteristics of the emotional environment should be recognized as distinct and important, and categorized. For individuals who are low in emotion differentiation, the characteristics of the events they attend to are not very specific, and therefore not uniquely associated with specific emotion labels. As such, informing individuals about the different characteristics of emotional events that can be considered as important, and showing examples of different ways to categorize these different aspects, can potentially increase individuals’ emotion knowledge.

Knowledge about emotion characteristics is referred to as emotion knowledge: the more of this information is available to the individual, the higher their emotion knowledge is. However, there appear to be individual differences in emotion knowledge, meaning that individuals differ in terms of how much or what type of knowledge they have about emotions (e.g., Bennett et al., 2005; Izard et al., 2008; Schlegel and Scherer, 2018). Importantly, an extensive study by Schlegel and Scherer (2018) found that emotion knowledge correlated with emotional understanding, emotion management, emotion recognition both in the self and in others, and cognitive skills, such as problem-solving, memory, and reasoning (Schlegel and Scherer, 2018), while another study showed that emotion knowledge was correlated with academic performance and social competence in young children (Izard et al., 2001, 2008). Together, these studies suggest that emotion knowledge may be important and beneficial for well-being because it can positively influence how individuals experience emotions and adaptively apply their emotion-related abilities.

In line with this past research, it has been theorized that this conceptual information on emotions and their components is constitutive and would end up making multimodal emotional instances more distinctive by adding complexity to their features (Barrett et al., 2001). It forms the base for how individuals process, communicate and deal with their own emotions (Barrett et al., 2001; Izard et al., 2011; Kashdan et al., 2015). Specifically, it has been argued that emotion knowledge is important for emotion differentiation, because conceptually knowing the different characteristics associated with the different emotions might enable individuals to better recognize these characteristics in themselves and will make differences and similarities between emotions more salient. This in turn might result in a more context-sensitive way of labeling emotions, and thus in higher emotion differentiation. However, empirical research on this relationship is lacking.

The current study is part of a larger study pre-registered at https://osf.io/j389k. Existing empirical research on emotion knowledge and theoretical literature on emotion differentiation implies that more conceptual knowledge of emotions should lead to higher levels of emotion differentiation. The previous studies showed that emotion differentiation could be changed. They provide evidence of emotion differentiation being malleable and variable over time rather than being a stable characteristic or personal trait. For instance, stress on 1day was negatively related to the level of negative emotion differentiation on a next day (Erbas et al., 2018). Moreover, Mindfulness-based intervention (MBI) led to improvement in both positive and negative emotion differentiation (Van der Gucht et al., 2018). In the current study, we examined this causal relationship between emotion knowledge and emotion differentiation empirically. More specifically, we increased emotional knowledge through an emotion knowledge intervention and assessed whether this increases individuals’ level of emotion differentiation, compared to a similar control condition that did not involve emotion-relevant knowledge. We had hypothesized that complementary information on emotions might help individuals to better identify their emotional experiences and navigate among them.

In order to examine the effect of emotion knowledge on emotion differentiation, we conducted an experimental study consisting of two conditions: in the experimental condition, participants received information about emotions through an emotion knowledge intervention, while in the control condition, participants received emotion-irrelevant information regarding countries and continents in order to take into account the Hawthorne effect (Phakiti, 2015). Emotion differentiation was assessed at three occasions: pre-intervention (T1), post-intervention (T2), and at follow-up (T3; 1month after T2). Emotion differentiation was considered separately for positive and negative emotions. We expected the emotion knowledge intervention to lead to an increase in emotion differentiation both at the between-person level and at the within-person level. More specifically, we expected (H1) participants in the experimental condition to have higher levels of emotion differentiation compared to the participants in the control group at T2 (between-participant level); and (H2) participants in the experimental condition to have a larger increase in emotion differentiation from T1 to T2 compared to the participants in the control condition. Finally, exploratively, we examined whether intervention effects were still present at T3.

## Materials and Methods

### Participants

Participants were recruited *via* the online participant platform Prolific (Palan and Schitter, 2018). A prescreening criterion on Prolific was set to show the study only to individuals whose first language is English. The number of participants was based on an *a priori* conducted power analysis with the software program G*Power (Faul et al., 2007) and on the recommendation to use at least 50 participants per group (Simmons et al., 2013). Power is smaller for interactions, so a power analysis was calculated for an interaction effect. The power analysis (ANOVA: Repeated measures, within-between interaction effect) to detect small effect size (*f*=0.15, *α*=0.05, with power 0.9, number of groups=2, number of measurements=3) indicated that at least 96 participants were needed. Due to the expected dropout between post-assessment and follow-up, the final sample (for pre- and post-assessment) consisted of 120 English-speaking participants (62 men and 58 women). We randomly assigned 60 individuals per condition with 31 men and 29 women in the control condition and 31 men and 29 women in the experimental condition. Participants were aged between 18 and 74years old (*M*=35.67, *SD*=13.43). Among them, 86.67% were White, 8.33% were Asian, 4.17% were Black, and 0.83% reported having a different ethnicity. English was the first language for 98.33% of participants with one participant having Lithuanian as their first language. With regard to marital status, 37.5% of the participants reported being single (never married), 33.33% were married, 18.33% were living together with a partner, and 10.84% had a different marital status. In terms of residency, 75.83% were residing in the United Kingdom, 10.83% were residing in the United States, and 13.34% elsewhere. Regarding the highest level of education completed, 33.33% of participants had a Bachelor’s degree, 30.83% completed some college but did not have a degree, 15.83% held a high school degree or equivalent, and 20.01% had other types of education. In addition, at the beginning of the study, 39.17% were employed full-time, 15.83% were employed part-time, 11.67% were students, and 33.33% had a different employment status.

Participants received a reward of £15 if they completed all parts of the study. The reward consisted of two payments and one bonus. Participants could only receive the first payment if they had completed the first 7days of the study. They received the second payment and the bonus when they completed the follow-up questionnaires. This study was approved by the Social and Societal Ethics Committee of University of Leuven, KU Leuven, Belgium (G-2017 12 1040).

### Materials

#### Emotion Differentiation

Emotion differentiation was measured with the Scenario Rating Task (SRT; Reisenzein and Hofmann, 1993; Schimmack and Diener, 1997) modified for Dizén and Berenbaum (Dizén and Berenbaum, 2011; Boden et al., 2013). The SRT measures participants’ emotional reactions in response to emotional scenarios, which depict real life events. Those scenarios were chosen as a standardized and previously used approach to model a situation, in which an emotion is likely to be experienced. The SRT comprised 20 scenarios (10 positive and 10 negative) depicting everyday life events, and each scenario is approximately 50–90 words long.

Participants rated the intensity of emotions they could feel in response to each scenario on a 7-point Likert scale ranging from 0 (“not at all”) to 6 (“extremely strongly”). There were 12 emotions to match the scenarios, mostly based on the six emotion categories (LOVE: love; JOY: joy; ANGER: anger, disgust; SADNESS: sadness, loneliness; FEAR: fear, anxiety; SHAME: shame, guilt) by Diener et al. (1995). Two emotions (relief and satisfaction) were added to the list: relief – to match the scenarios from the SRT, and satisfaction, as an alternative to contentment in Diener et al. (1995), as contentment was absent in an emotion database used to create the emotion knowledge intervention (described below). An emotion differentiation index was computed for each participant by calculating the average intra-class-correlation coefficient (ICC) measuring consistency separately among eight negative (negative emotion differentiation) and four positive (positive emotion differentiation) emotions across 20 different scenarios (Shrout and Fleiss, 1979). Since reliable ICCs lie between 0 and 1, we excluded one negative value (Giraudeau, 1996). Similar to the previous research (Kalokerinos et al., 2019), we normalized ICCs by applying a Fisher’s r to z transformation. In order to have more intuitive output, we reverse-scored normalized ICCs (−1×ICC), so that higher scores indicated higher differentiation.

#### Emotion Knowledge Intervention

**Figure 1:**
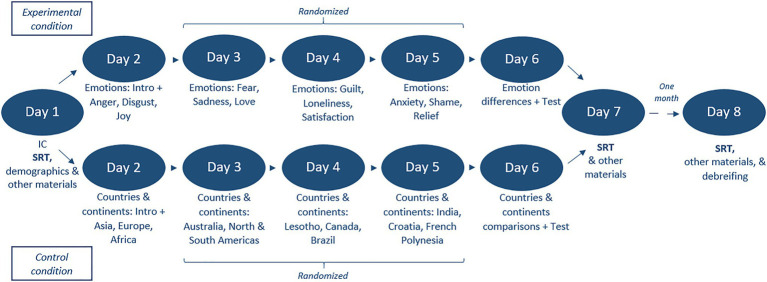
Flowchart of the study procedure.

Participants were randomly assigned to two conditions: the experimental condition in which participants received information about emotions in order to increase their conceptual emotion knowledge, and the control condition in which participants received information about an unrelated topic (i.e., continents and countries). The intervention lasted for 5 consecutive days and took place between days 2 and 6 of the study (see Figure 1): On the first 4days of the intervention (days 2–5 of the study) participants received the information regarding emotions or countries/continents concept by concept (three per day) and on the 5th day (day 6 of the study), they received information on those concepts in comparison to one another. The order of the emotion concepts of days 3–5 was randomized (day 2, the first day of the intervention, was not part of the randomization, meaning that everyone received the same information on that day, because it also contained general information about the study, and therefore it was logistically not possible to make it part of the randomization). In the experimental condition, participants were instructed to study information on 12 different emotions. The list of these 12 emotions is the same as in the SRT and is mainly based on Diener et al. (1995): love, joy, satisfaction, relief, anger, disgust, sadness, loneliness, fear, anxiety, shame, and guilt. For each emotion, participant received a text description and visual stimuli (the materials can be shared upon request to the corresponding author). Text description of emotions included a “definition” of a certain emotion as well as circumstances and situations in which individuals might experience this emotion (Delft Institute of Positive Design, n.d., 2017; Desmet, 2012; Yoon et al., 2015). The materials that we used were retrieved from the Delft Institute of Positive Design database.

Participants were also presented with three visual stimuli for each emotion. These stimuli were retrieved from the tool and methods database of the Delft Institute of Positive Design (Delft Institute of Positive Design, n.d., 2017; Yoon et al., 2015; Kurdi et al., 2017). For each emotion, the stimuli consisted of one drawing and two photos, and each stimulus had a different function. Either it was aimed at eliciting a certain emotion (a photo), or it depicted a person who was experiencing this emotion (a drawing and a photo). For each emotion, in order to provoke more in depth thinking, participants were asked “What situations might make you feel [emotion]?” After all emotions had been presented, on the 5th day of the intervention, participants received information regarding the differences between emotions (e.g., how do fear and anxiety differ, in which situations do each of them occur).

In the control condition, participants studied six continents (Africa, Asia, Australia and Oceania, Europe, North America, and South America) and six countries (Brazil, Canada, Croatia, French Polynesia, India, and Lesotho). The information regarding these countries and continents was retrieved from the Wikipedia (n.d.) and was presented in such a way that it was very similar to how the materials were presented in the emotion knowledge intervention. The text descriptions contained the geographical information of the country or continent. For each country or continent, there were three neutral visual stimuli also retrieved from the Wikipedia (n.d.). The visual stimuli comprised a map of the country or continent, a flag of the country and a satellite view of the continent, as well as a landscape shot of the indicated area. Afterward, in order to make the materials of the control condition similar to the materials of the experimental condition, participants were asked the question “Would you like to visit [country/continent]? Why or why not?”

On the last day of the intervention, participants received materials to learn more about the differences and similarities between countries/continents (e.g., differences or similarities in their population density, territory, and climate).

Materials for both conditions were made very similar to each other by implementing the same number of items (12 emotions or 12 countries and continents), the same structure, and the same number of pictures. All information regarding a certain concept was presented on the same page. In order to enhance attention to materials, in both conditions, participants were informed at the start that at the end of the intervention, they would be offered a test on the materials they studied. At the end of the 5th day of the intervention (day 6 of the study), they were offered the test to complete.

Attention check items (Oppenheimer et al., 2009; Berinsky et al., 2014) were included in order to control the quality of the data. “Fair” attention check items recommended by Prolific were applied. We included both open-ended and close-ended items. An example of an open-ended item is: “The color test is simple, when asked for your favorite flower you must enter the word magnolia in the text box below. Based on the text you read above, what flower have you been asked to enter?” An example of the closed-ended item is: “It’s important that you pay attention to this study. Please tick ‘Strongly disagree.’” Four attention check items were included in the longer surveys (which were assessed on days 1, 7, and 8) and one attention check item was included in the short surveys (which were assessed on days 2–6). Across three time points, 98.58% of attention check items were answered correctly.

### Procedure

The study was created and edited on the Qualtrics Survey Platform and then conducted with Prolific – a participant pool for online experiments (Palan and Schitter, 2018). There is evidence that data recruited *via* crowd-working platforms is of good quality (Buhrmester et al., 2011).

The experiment required participation for 8 days. At pre-intervention (T1; approximately 30 min long) on day 1 participants signed informed consent and completed the SRT and other tasks and questionnaires for a larger project. Among all tasks and questionnaires, participants completed the SRT first, right after the informed consent. A known problem with online participant pools is that instead of a “real” participant, sometimes questionnaires are completed by bots (e.g., Teitcher et al., 2015; Bai, 2018). Therefore, in order to ensure that our participants were real persons and not bots, on the 1st day of the study, participants were asked to answer an open question (“what is your favorite dish?”) in two full sentences. In case a participant had given a nonsensical answer, we would have not invited them to the following steps of the study. However, this was not the case for any of the participants; therefore, all participants were invited for the intervention part of the study. To control bots further in the study, the Completely Automated Public Turing test to tell Computers and Humans Apart (CAPTCHAs; von Ahn et al., 2003) were included into all 8 days of the study. On days 2–5 (approximately 15 min each) participants received information about emotions (emotion knowledge intervention in the experimental condition) or countries (control condition) one by one, with three concepts per day (e.g., anger, disgust, and joy; or Asia, Europe, and Africa). The sequence of concepts was randomized for days 3–5. On day 6 participants studied emotions (or countries) in comparison to each other and completed a test based on the received knowledge. On day 7 (T2, post-intervention) and 1 month after (T3, follow-up) participants completed the same questionnaires and tasks again (approximately 30 min each day). At follow-up (T3), which was 1 month after T2, participants additionally received the debriefing.

## Results

### Data-Analytic Strategy

To test our hypotheses regarding emotion differentiation, we applied two mixed ANOVAs per hypothesis. Because of the drop out, we first compared T1 and T2 with a full sample and then separately we compared T1, T2, and T3 with a smaller sample. Thus, 2 (Time; within-factor)×2 (Condition; between-factor) mixed ANOVA with the full sample of 120 participants (60 per condition) was conducted to compare T1 and T2. Then, an additional exploratory analysis of 3 (Time; within-factor)×2 (Condition; between-factor) mixed ANOVA with 103 participants (53 of them from the experimental condition) was conducted to make a comparison across T1, T2, and T3. Scenarios from the SRT were used to compute positive and negative emotion differentiation indices. Both hypotheses H1 (between-person level) and H2 (within-person level) were tested separately for positive and negative emotion differentiation. SPSS version 26.0 (IBM Corp., 2019) was used for data analysis.

### Hypotheses Testing

We conducted one-way ANOVAs on 120 individuals to compare emotion differentiation in participants of both conditions at T1. There was no effect of condition for negative emotion differentiation, *F*(1,118) < 0.01, *p*=0.977, *η_p_*^2^ < 0.001, or for positive emotion differentiation, *F*(1,118)=1.02, *p*=0.316, *η_p_*^2^=0.009, so individuals from both conditions had approximately the same levels of emotion differentiation before the intervention.

#### Positive Emotion Differentiation

A mixed 2×2 ANOVA was run. There was an effect of time, *F*(1,118)=14.47, *p* < 0.001, *η_p_*^2^=0.109, observed power of 0.97, with participants having higher positive emotion differentiation at T2 (*M*=−1.16, *SD*=0.42) than at T1 (*M*=−1.28, *SD*=0.35). There was no effect of condition, *F*(1,118)=3.00, *p*=0.086, *η_p_*^2^=0.025, observed power of 0.40, meaning that participants in the experimental condition did not differ significantly in positive emotion differentiation from the participants in the control condition. However, there was no interaction between time and condition, meaning that the emotion knowledge manipulation did not produce any significant changes in the experimental condition as compared to the control condition, *F*(1,118)=2.13, *p*=0.147, *η_p_*^2^=0.018, observed power was 0.30.

**Figure 2:**
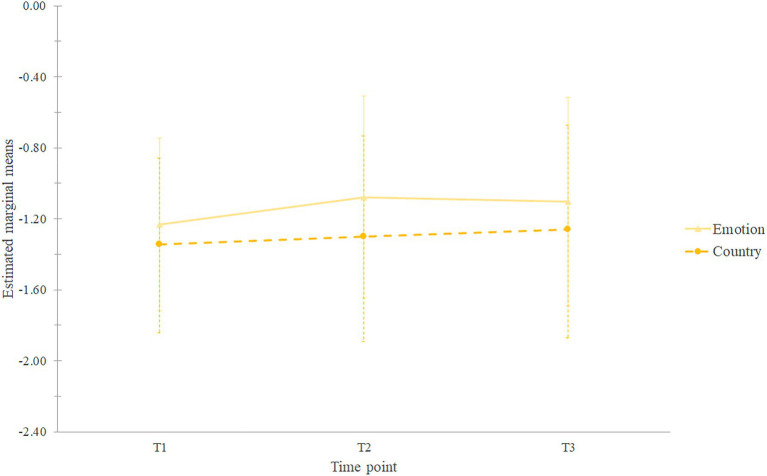
Estimated marginal means of positive emotion differentiation. Error bars represent standard deviations.

In order to exploratively compare T1, T2, and T3, we applied a mixed 3×2 ANOVA with 103 participants (Figure 2). There was an effect of time, *F*(2;202)=5.29, *p*=0.006, *η_p_*^2^=0.050, observed power of 0.83. *Post-hoc* pairwise comparisons revealed that individuals had higher positive emotion differentiation at T2 (*M*=−1.19, *SD*=0.41) than at T1 (*M*=−1.30, *SD*=0.38, *p*=0.011), T2 did not differ significantly from T3 (*M*=−1.18, *SD*=0.34, *p*=1.000), but T1 did (*p*=0.029). There was an effect of condition, *F*(1,101)=6.44, *p*=0.013, *η_p_*^2^=0.060, observed power of 0.71, with individuals in the emotion condition having higher positive emotion differentiation (*M*=−1.14, *SD*=0.46) than participants in the country condition (*M*=−1.30, *SD*=0.48). Contrary to both our hypotheses, there was no interaction between time and condition.

In conclusion, although positive emotion differentiation appeared to improve with time and in the emotion condition, the interaction between time and condition did not reach the significance level. Therefore, our hypotheses (H1 and H2) were not confirmed.

#### Negative Emotion Differentiation

A mixed 3×2 ANOVA was run on 120 individuals. There was a main effect of time, *F*(1,118)=18.49, *p* < 0.001, *η_p_*^2^=0.135, observed power of 0.99, with people having higher negative emotion differentiation at T2 (*M*=−1.28, *SD*=0.41) than at T1 (*M*=−1.39, *SD*=0.36). The effect of condition was significant, *F*(1,118)=4.03, *p*=0.047, *η_p_*^2^=0.033, observed power of 0.51: individuals from the experimental condition (*M*=−1.27, *SD*=0.50) had higher differentiation than individuals from the control condition (*M*=−1.40, *SD*=0.50). There was an interaction between time and condition, *F*(1,118)=24.85, *p* < 0.001, *η_p_*^2^=0.174, observed power of 0.99, meaning that participants in the experimental condition improved their negative emotion differentiation more than participants in the control condition. Since the interaction was significant, we conducted analyses of simple main effects, which revealed that there was an effect of time for the experimental group, *F*(1,59)=37.69, *p* < 0.001, *η_p_*^2^=0.390 with participants having higher negative emotion differentiation at T2 (*M*=−1.15, *SD*=0.55) compared to T1 (*M*=−1.39, *SD*=0.46). However, that was not the case for the control group, *F*(1,59)=0.27, *p*=0.603, *η_p_*^2^=0.005. This indicates that our within-person hypothesis (H2) was confirmed, meaning that in the experimental condition, negative emotion differentiation improved, but in the control condition, it did not improve. As mentioned above (one-way ANOVA), the analysis of simple main effects confirmed that there was no effect of condition for T1, *F* (1,118)=0.01, *p*=0.977, *η_p_*^2^ < 0.001. However, there was an effect of condition for T2, *F* (1,118)=12.46, *p*=0.001, *η_p_*^2^=0.095, with people from the experimental condition having higher negative emotion differentiation (*M*=−1.15, *SD*=0.57) than people from the control condition (*M*=−1.41, *SD*=0.57). This indicates that our between-person hypothesis (H1) was also confirmed, meaning that before the intervention, the difference in negative emotion differentiation was not significant between the groups. However, after the intervention, the level of negative emotion differentiation was significantly higher in the experimental group than in the control group.

**Figure 3:**
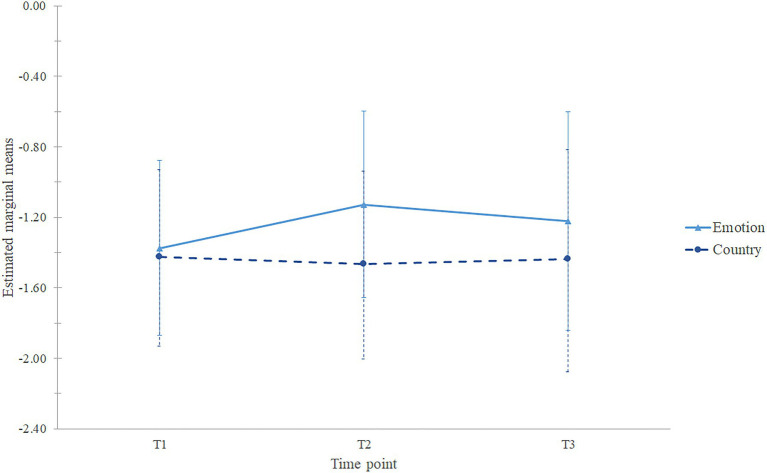
Estimated marginal means of negative emotion differentiation. Error bars represent standard deviations.

In order to exploratively compare T1, T2, and T3, we applied a mixed 3×2 ANOVA (Figure 3) with 103 effective data points (54 participants in the experimental condition, and 49 participants in the control condition). The test of sphericity was significant (*p* < 0.001) and larger than 0.75 (Greenhouse–Geisser *ε*=0.87), therefore we used Huynh-Feldt correction. There was an effect of time, *F*(1.79,181.24)=6.53, *p*=0.003, *η_p_*^2^=0.061, observed power of 0.88. *Post-hoc* pairwise comparisons revealed that individuals had higher negative emotion differentiation at T2 (*M*=−1.30, *SD*=0.38) than at T1 (*M*=−1.40, *SD*=0.36, *p*=0.001), T3 (*M*=−1.33, *SD*=0.44) did not differ significantly from T1 (*p*=0.122) and T2 (*p*=0.633). An effect of condition was also significant, *F*(1,101)=8.33, *p*=0.005, *η_p_*^2^=0.076, observed power of 0.82, meaning that the experimental group was higher in negative emotion differentiation (*M*=−1.24, *SD*=0.50) than the control group (*M*=−1.44, *SD*=0.51). There was an interaction between time and condition, *F*(1.79,181.24)=11.80, *p* < 0.001, *η_p_*^2^=0.105, observed power was 0.99, meaning that participants from the experimental condition improved their negative emotion differentiation more than people from the control condition. Since the interaction was significant, we conducted analyses of simple main effects, which revealed that there was indeed an effect of time for the experimental group, *F*(1.86,96.73)=21.37, *p* < 0.001, *η_p_*^2^=0.291 with participants having higher negative emotion differentiation at T2 (*M*=−1.13, *SD*=0.53) compared to T3 (*M*=−1.22, *SD*=0.57) and T1 (*M*=−1.38, *SD*=0.51). *Post-hoc* tests revealed that the level of negative emotion differentiation significantly differed between all three time points: T1 from T2 (*p* < 0.001), T1 from T3 (*p*=0.002), and T2 from T3 (*p*=0.013); it increased from T1 to T2 and decreased from T2 to T3, however at T3 it was still higher than at T1. There was no effect of time for the control group, *F*(1.63,79.85)=0.39, *p*=0.636, *η_p_*^2^=0.008, meaning that the level of negative emotion differentiation was not different between the three assessment points for the participants in the control condition. This means that our within-person hypothesis (H2) was confirmed: in the experimental condition, negative emotion differentiation improved, but in the control condition, it did not. As mentioned above, there was no effect of condition at T1, but there was at T2. A one-way ANOVA showed that there was an effect of condition also at T3, *F*(1,101)=6.08, *p*=0.015, *η_p_*^2^=0.057, which demonstrated that individuals from the experimental condition had higher negative emotion differentiation (*M*=−1.22, *SD*=0.62) than individuals from the control condition (*M*=−1.43, *SD*=0.64) at follow-up. This means that our between-person hypothesis (H1) was confirmed: meaning that before the intervention, the difference in negative emotion differentiation was not significant between the groups, but after the manipulation, the level of negative emotion differentiation was significantly higher in the experimental group than in the control group. Moreover, at follow-up, these differences were still significant, implying that the effect was lasting.

In conclusion, both between- (H1) and within-person (H2) hypotheses were confirmed for negative emotion differentiation, providing evidence for the improvement of differentiation due to the emotion knowledge intervention.

## Discussion

In the past years, a large number of studies have shown that lower levels of emotion differentiation are related to maladaptive outcomes. However, there is very limited evidence on the direction of this relationship. The current study is among the first to show experimentally that emotion differentiation abilities can be improved through an intervention. We hypothesized that increasing individuals’ emotion knowledge would help them to better differentiate between emotions and found that the individuals who received the emotion knowledge intervention indeed improved their levels of emotion differentiation. Specifically, we found that the emotion knowledge intervention benefited emotion differentiation on both the within- and between-person level. Compared to the control group, we found that the level of negative emotion differentiation had increased significantly from baseline to post-intervention. In other words, for negative emotions, we found that the intervention increased the level of emotion differentiation, whereas there was no significant increase in the control group. Moreover, at post-intervention, the level of differentiation was significantly higher in the experimental group compared to the control group. The effect size for interaction at T2 for negative emotion differentiation was 0.174, *η_p_*^2^=0.174, indicating medium effect according to the rule of thumb (MRC CBU, n.d.). Importantly, this effect was still visible at the third measurement occasion, which was a month after the end of the intervention. This indicates that the effects of the intervention did not just cause a momentary increase, but that these changes were lasting (at least for a month). In sum, with regard to negative emotions, this study provides a clear direction of the effect and thus inputs to the directional literature on the topic of emotion differentiation. Considering the fact that some individuals appear to have low levels of emotion differentiation, which in turn is associated with negative outcomes such as depression, negative emotions, and maladaptive behavior (Kashdan et al., 2010; Lennarz et al., 2018; Willroth et al., 2019), improving emotion differentiation might be a promising way forward to increased well-being.

With regard to positive emotion differentiation, changes were observed in both conditions, which indicate that the increase in emotion differentiation was not due to the emotion knowledge intervention. There can be different reasons of these findings. First, maybe the mere participation in the experiment (e.g., De Vuyst et al., 2019) has increased the level of positive emotion differentiation: the participants repeatedly completed questionnaires about emotions and well-being (which were the part of a larger study; e.g., mindfulness, self-esteem, and depression scales) and the Scenario Rating Task, which might have caused them to think in more detail about their emotions overall, including positive ones. However, this effect was not observed for negative emotion differentiation (i.e., negative emotion differentiation did not increase in the control condition, instead there was an interaction between time and condition); therefore, this explanation is not very likely. Another possible reason is that the positive emotion differentiation index was less reliable than the negative emotion differentiation index, because it only consisted of four emotions, whereas the negative differentiation index consisted of eight emotions. Moreover, we included joy as one of positive emotions; however, it could be an umbrella term for positive emotions overall (Sauter and Scott, 2007; Lyubomirsky and Kurtz, 2009; Sauter, 2017; Shiota, 2017). Therefore, participants might have picked up joy as an experienced emotion instead of going into details and report relief or satisfaction. The absence of an emotion knowledge intervention effect for positive emotion differentiation may be also due to the fact that negative emotions are more necessary for the survival than positive emotions from an evolutionary point of view (Buss, 2000), and thus individuals are more motivated to improve their differentiation of negative emotions. For example, if an individual cannot differentiate sadness and fear, they might get into difficulties: if it is sadness, active actions might not be needed, but if it is fear, this individual should take actions to save their life. However, if someone cannot differentiate admiration from interest, that is much less likely to lead to detrimental consequences. Thus, although negative emotions may sometimes be considered as undesirable, they have an important function, which, for example, is highlighted in existential positive psychology (e.g., Wong and Hwang, 2021). People’s lives consist not only of pleasant events, and the ability to deal with negative emotions appears to be an adaptive strategy (e.g., Diener and Seligman, 2002; Wong and Bowers, 2018; Wong et al., in press).

Overall, this study shows that increasing individuals’ level of emotion knowledge can increase the level of negative emotion differentiation. This finding is important since it allows for a more directional test of the relationship between emotion differentiation and indicators of well-being in later studies. Furthermore, the current intervention, or components of this intervention, can be applied in the context of psychotherapy or clinical interventions to increase the level of emotion differentiation by making people more aware of the components of and the differences between emotions.

Apart from this practical implication, the findings from this study also have a theoretical implication. Previous research showed that there are several pathways through which emotion differentiation can be influenced. One pathway is thought to be through information processing: since stress on 1day predicted emotion differentiation on the next day (Erbas et al., 2018), individuals’ knowledge and/or perception of the environment as more or less stressful determined their ability to differentiate emotions. A second pathway is thought to be through attention: Van der Gucht et al. (2018) showed that a mindfulness-based intervention led to an increase in differentiation of positive and negative emotions. Being mindful refers to drawing novel distinctions (Langer, 1989), and in order to do so, one should be attentive to their environment, which could include their emotional state, enabling individuals to pay more attention to their emotional experience. The current findings suggest a third pathway, namely through conceptual emotion knowledge, where more knowledge about emotions appears to increase the level of emotion differentiation. Discovering new pathways allows for a better understanding of what emotion differentiation is and how it can be changed, which opens the doors to better and more effective interventions for the clinic. Furthermore, since emotion knowledge appears to influence individuals’ tendency to differentiate between emotions, it may also improve emotional intelligence (Salovey and Mayer, 1990; Mayer et al., 2004) especially its emotion understanding branch. Previous research shows that negative emotion differentiation as part of the emotional complexity construct, appears to be related only to emotion understanding (and not to emotion intelligence overall), but this relationship was not significant anymore after controlling for negative affect (MacCann et al., 2020). Although the relationship between emotion differentiation and emotion intelligence appears fragile, emotion knowledge may still have an influence on other emotional intelligence branches, for example, on emotion perception (i.e., the ability to perceive emotions in the environment) or emotion facilitation (i.e., the ability to use emotions to generate thought). Future research may further examine those relationships.

In terms of future directions, it might be important to set up an intervention with a more equal number of positive and negative emotions. This will not only help to capture the effect of emotion knowledge on positive emotion differentiation more extensively, but it will also allow to compare between the effects of the emotion knowledge intervention on differently valenced emotions. While negative emotions may be more relevant for focusing and narrowing attention, positive emotions may broaden individuals’ thought repertoires (Fredrickson, 2001; Fredrickson and Branigan, 2005). However, the scope of emotion differentiation research has been mostly on negative emotions, therefore examining positive emotion differentiation more extensively in future research is pertinent.

Furthermore, it is important to assess whether the current findings extend to other populations. The current study only included healthy individuals who may not particularly be in need of developing higher levels of emotion differentiation. However, individuals with for instance major depressive disorder or borderline personality disorder tend to have lower levels of emotion differentiation (Suvak et al., 2011; Demiralp et al., 2012) and might therefore benefit more from an intervention.

Furthermore, the sample of the study was not representative, since most participants reported to reside in the United Kingdom. The intervention might have different effects in other countries since there are cultural emotion differences (e.g., Boiger et al., 2018). For instance, differences can be found in behavioral and physiological aspects of emotions, with Easterners having fewer physiological activity than Westerners and Westerners experiencing emotions more actively (with higher arousal; reviewed by Lim, 2016). If individuals from different cultures are different in emotion experience and expression, they might be also different in the perception of emotion knowledge. Therefore, in order to generalize the current findings, a more culturally diverse population may be needed.

In addition, about a third of the participants completed the follow-up assessment in March 2020, when the COVID-19 pandemic had started, those circumstances were likely to affect participant’s performance. Furthermore, as mentioned in the “Introduction” section, the reported study was part of a larger project that included more measures of emotional complexity (e.g., emodiversity) and well-being (e.g., depression), because we were interested in how the emotion knowledge intervention and its effect on emotion differentiation would relate to those measures. However, the findings for these other variables were very inconsistent (though the well-being measures were trending in the right direction), and it is unclear whether this was caused by the pandemic, or by other factors.

Finally, while the current intervention was successful in increasing the level of emotion differentiation, it is possible that a more personal and intensive intervention might be even more effective. For instance, the current study was conducted online, and there was no personal interaction between the researcher and the participants. Moreover, while the participants were explicitly instructed to pay attention to the materials that were presented as part of the intervention, these materials were presented online and it is possible that perhaps not all participants were equally motivated to learn all information from the screen. Furthermore, the information was presented to the participants in a passive way, whereas it may also be important that individuals get the opportunity to practice and apply the information to the real world. Therefore, a longer in person intervention, which also includes interactions between the researcher/clinician and the participants, and practice sessions with feedback from the researcher/clinician, might potentially be more effective, and could result in more structural changes in emotion processing than the current intervention.

To conclude, increasing emotion knowledge by providing individuals with information about the definitions of emotions, the circumstances when those emotions are likely to emerge and showing them related pictures appear to be beneficial for negative emotion differentiation. Individuals with low negative emotion differentiation might therefore benefit from an emotion knowledge intervention to improve their ability to make finer distinctions among their emotions and thus subtracting more granular information from their environment.

## Data Availability Statement

The raw data supporting the conclusions of this article will be made available by the authors, without undue reservation.

## Ethics Statement

The studies involving human participants were reviewed and approved by the Social and Societal Ethics Committee of University of Leuven, KU Leuven, Belgium (G-2017 12 1040). The patients/participants provided their written informed consent to participate in this study.

## Author Contributions

EV, PK, and YE contributed to the conceptualization and design of the study. EV collected the data and performed the statistical analysis. EV wrote the first draft of the manuscript. PK and YE edited the manuscript. All authors have read and approved the submitted version.

## Funding

During the majority of this project, YE was supported by a postdoctoral fellowship of the Research Foundation Flanders (FWO).

## Conflict of Interest

The authors declare that the research was conducted in the absence of any commercial or financial relationships that could be construed as a potential conflict of interest.

## Publisher’s Note

All claims expressed in this article are solely those of the authors and do not necessarily represent those of their affiliated organizations, or those of the publisher, the editors and the reviewers. Any product that may be evaluated in this article, or claim that may be made by its manufacturer, is not guaranteed or endorsed by the publisher.
